# Autophagy-Related Gene *ATG7* Polymorphism Could Potentially Serve as a Biomarker of the Progression of Atrophic Gastritis

**DOI:** 10.3390/jcm13020629

**Published:** 2024-01-22

**Authors:** Naoyuki Yamaguchi, Takuki Sakaguchi, Miki Taira, Daisuke Fukuda, Ken Ohnita, Tatsuro Hirayama, Kazuo Yashima, Hajime Isomoto, Kazuhiro Tsukamoto

**Affiliations:** 1 Department of Gastroenterology and Hepatology, Nagasaki University Graduate School of Biological Sciences, 1-7-1 Sakamoto, Nagasaki 852-8501, Japan; 2 Department of Gastroenterology and Nephrology, Faculty of Medicine, Tottori University, 36-1 Nishi-cho, Yonago 683-8504, Japan; 3 Department of Pharmacotherapeutics, Nagasaki University Graduate School of Biomedical Sciences, 1-7-1 Sakamoto, Nagasaki 852-8501, Japan; 4 Department of Surgical Oncology, Nagasaki University Graduate School of Biological Science, 1-7-1 Sakamoto, Nagasaki 852-8501, Japan; 5 Fukuda Yutaka Clinic, 3-5 Hamaguchi-machi, Nagasaki 852-8107, Japan; 6 Shunkaikai Inoue Hospital, 6-12 Takara-machi, Nagasaki 850-0045, Japan

**Keywords:** *H. pylori*, SNP (single nucleotide polymorphism), *ATG7*

## Abstract

Cytotoxin-associated gene A (CagA) is an oncoprotein that *H. pylori* injects into the host’s gastric epithelial cells and that induces proinflammatory cytokines, such as interleukin (IL)-18 and IL-1β. As a result, it leads to atrophic gastritis (AG), a precancerous lesion of gastric cancer. On the other hand, host cells degrade CagA using autophagy systems. However, few studies exist about the single nucleotide polymorphisms (SNPs) in *MAP1LC3A*, *MAP1LC3B*, *ATG4A*, *ATG4B*, *ATG4C*, *ATG7*, and *ATG13,* which belong to the autophagy-related genes concerning AG. This study aimed to detect biomarkers associated with AG. Herein, *H. pylori*-positive subjects (*n* = 200) were divided into the AG (*n* = 94) and non-AG (*n* = 106) groups. Thirty tag SNPs were selected from the above seven candidate genes. The SNP frequency between the two groups was analyzed. The frequency of the C/T or T/T genotype at rs4683787 of *ATG7* was significantly lower in the AG group than in the non-AG group (*p* = 0.034, odds ratio = 0.535). Based on multivariate analysis, the C/C genotype of rs4684787 and age were independently associated with gastric mucosal atrophy. This finding helps stratify the patients needing timely endoscopic screening or early eradication of *H. pylori*.

## 1. Introduction

**Figure 1:**
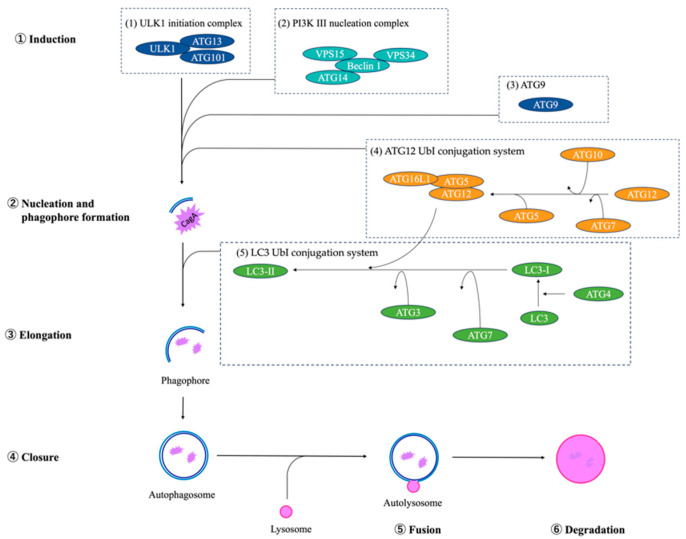
The process of autophagy in the extension stage has five reaction systems: (1) ULK1 initiation complex, (2) Pl3KIII nucleation complex, (3) ATG9, (4) ATG12 UbI conjugation system, and (5) LC3 UbI conjugation system. A ULK1 initiation complex, PI3KIII nucleation complex, and ATG9 are required for autophagy induction. The LC3 UbI conjugation system, in which ATG3 and ATG7 process LC3, engages with the autophagosome membrane. The ATG12 UbI conjugation system is needed both in the LC3 conjugation system and autophagy induction. The autophagosome combines with a lysosome, resulting in autolysosome formation, and degrades the cargo, such as CagA *H. pylori* injects.

Autophagy, which Christian de Duve named in 1963, is highly conserved in eukaryotes [1]. Autophagy is a degradation and recycling system for unwanted cytoplasmic material to maintain cellular homeostasis and functions and to make energy in response to various stress, such as nutrient deprivation, growth factor depletion, infection, and hypoxia [2]. The macroautophagic process, which is the predominant form, begins with forming a double-membrane compartment named a “phagophore”. The double membrane of the phagophore expands and isolates the components from the cytosol, which is named an “autophagosome”. Finally, the autophagosome fuses with a lysosome named autolysosome, and the cargo is degraded. In the extension stage, there are five reaction systems that are primarily associated with autophagosomes: the ULK1 initiation complex, Pl3KIII nucleation complex, ATG9, ATG12 UbI conjugation system, and LC3 UbI conjugation system (Figure 1) [3]. Autophagy-related (ATG) genes strictly regulate the sequence of autophagy, and recent studies have shown that the defect of autophagy is associated with several diseases [4,5,6]. In addition, Iris Grosjean et al. reported that a total of 263 single nucleotide polymorphisms (SNPs) within ATG genes have been found to exhibit associations with 117 distinct autoimmune, inflammatory, cardiovascular, neurological, and pulmonary diseases and phenotypic traits. [7]. In the field of gastric carcinoma (GC), which is developed eventually from atrophic gastritis (AG), *ATG3*, *ATG4B*, *ATG4C*, *ATG5*, *ATG7*, *ATG10*, *ATG12*, *ATG16L1*, and *TECPR1* mRNA levels were associated with the overall survival of GC [8].

Gastric mucosal atrophy is defined as the “loss of proper gastric glands” in a histologic definition [9] and as a preneoplastic condition mainly caused by chronic infection with *Helicobacter pylori* (*H. pylori*). AG’s progression risk to gastric adenocarcinoma is estimated to range from 0.1% to 0.3% per year, or 1–3% of infected individuals develop GC [10,11], and is known to increase with the severity of AG [12]. Even after eradication therapy, the cancer risk after eradication depends on the extent of the AG before eradication and is highly correlated with the severity of corpus atrophy [13]. In *H. pylori*-related AG, the area of atrophy progresses from the antrum toward the corpus along the lesser curve [13,14]. It is generally known that AG is usually severe in elderly patients in proportion to the duration of *H. pylori* infection [15]. However, while some adult patients infected with *H. pylori* do not have severe AG, some children or infants have severe AG [16,17]. Therefore, we hypothesize that age and some host factors would affect the extension of AG.

We have reported that the G/G genotype of rs6431659 in *ATG16L1* could be a good biomarker for detecting future severe atrophic gastritis [18]. However, the association between the SNPs of the *ATGs* involved in the LC3 conjugation system and AG was not elucidated. In this study, among the genes essential to autophagy involved in autophagosome formation, *microtube-associated protein 1 light chain 3 alpha* (*MAP1LC3A*) and *micro-tube-associated protein 1 light chain 3 beta* (*MAP1LC3B*), which are mammalian homologs of *LC3*, as well as *ATG4A*, *ATG4B*, *ATG4C*, *ATG7*, and *ATG13*, were deemed candidate genes for susceptibility to AG in *H. pylori*-infected individuals and correlation analyses were performed between the SNPs in the candidate genes and the progression of AG. Our purpose in this study is to detect biomarkers involved in the progression of atrophic gastritis.

## 2. Materials and Methods

### 2.1. Subjects

As previously reported [18,19,20], 503 participants who underwent upper gastrointestinal endoscopy at Fukuda Surgical Hospital for their medical checkup were included. Two hundred were diagnosed as *H. pylori*-positive based on the *H. pylori* antibody titer. After that, we classified the participants into the AG and non-AG groups based on the pepsinogen (PG) method. Specifically, participants with a PG I ≤ 70 and a PG I/II ratio ≤ 3.0 were assigned to the AG group, and those who failed to meet either criterion were assigned to the non-AG group [19]. All of them signed an informed consent form. The Human Genome Genetic Research Institutional Review Board of Nagasaki University approved the present study.

### 2.2. Method

We obtained peripheral blood from all the participants, and DNA was extracted according to the protocol for NucleoSpin Blood^®^ (Takara, Kusatsu, Japan). We measured the concentration of the extracted DNA using a Nanodrop^®^ UD-1000 (Nanodrop Technologies, Wilmington, DE, USA), and each extracted DNA was diluted with a Low TE buffer (10 mM Tris·HCl [pH 8.0] and 0.1 mM EDTA) to obtain a final 10 ng/μL concentration.

**Figure 2:**
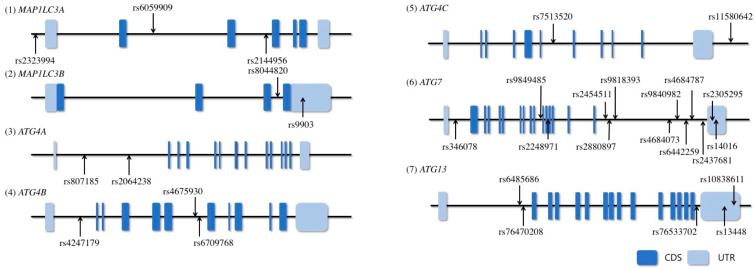
Gene structures and locations on the genotyped tag SNPs in each gene. Horizontal lines indicate the base sequence of each gene, and boxes indicate exons. The arrows point to the positions of the genotyped tag SNP loci in this study. CDS, coding sequence; UTR, untranslated region.

As mentioned in the introduction, we defined *MAP1LC3A*, *MAP1LC3B*, *ATG4A*, *ATG4B*, *ATG4C*, *ATG7*, and *ATG13* as candidate genes. We extracted tag SNPs from the candidate genes as follows. First, all the SNPs within the candidate gene and 3 kb upstream from the promoters, reported by the 1000 Genome Project database (GRCh37 p.13) in Japanese individuals, were selected. Second, we excluded the SNPs with minor allele frequencies < 0.1. Subsequently, we selected SNPs that met r2 > 0.8 (pair-wise tagging method) as tag SNPs using the Haploview 4.2 software. Figure 2 shows the locations of the tag SNPs in the candidate genes.

Next, the selected tag SNPs were analyzed using the polymerase chain reaction (PCR)-high-resolution melting (HRM) analysis method or PCR-restriction fragment length polymorphism (PCR-RFLP) or PCR-direct DNA sequencing method. The primers for the PCR were designed to include the respective tag SNPs. The primer sequences, annealing temperatures, and cycle numbers used in the analysis are presented in Supplementary Table S1. The detailed protocols for the three methods are described below.

Clinical characteristics between the AG and non-AG groups were compared using the Mann–Whitney U or chi-square tests. The chi-square test was used for the polymorphism analysis to determine whether each SNP satisfied the Hardy–Weinberg equilibrium (HWE). The chi-square or Fisher’s exact test was conducted to compare the allele and genotype frequency with each of three genetic models (allele, the minor allele dominant, and the minor allele recessive). Since the average age of the AG group was older than that of the non-AG group, SNPs significantly different in the univariate analysis were subjected to multivariate analysis with age to verify the independence between age and genotype. The statistical analyses were performed using SNPAlyze 7.0 (Dynacom Co., Ltd., Yokohama, Japan) for the chi-square and Fisher’s exact test to compare the frequency of the alleles and genotypes. IBM SPSS 20.0 (IBM Japan, Tokyo, Japan) and GraphPad Prism 5 (GraphPad Software, Inc., La Jolla, CA, USA) were employed to perform the Mann–Whitney U and chi-square test for multivariate logistic regression analysis to verify the independence between age and genotype, as well as for comparing the clinical characteristics between the two groups. The odds ratio (OR) and 95% confidence interval (95%CI) were calculated, and *p* < 0.05 was deemed significant.

#### 2.2.1. PCR-High Resolution Melting (HRM) Analysis with a Non-Labeled Probe Method

The respective polymorphic regions were amplified via PCR using the GeneAmp PCR System 9700 (Life Technologies, Carlsbad, CA, USA) or the T100 Thermal Cycler (Bio-Rad, Hercules, CA, USA). A mixture of genome DNA (10 ng), 1X Go Taq^®^ Colorless Master Mix, forward primer (0.06 μM), reverse primer (0.3 μM), probe (0.3 μM), and SYTO9 (2 μM; Life Technologies) was prepared to attain a PCR reaction fluid volume of 20 μL (Tables S1 and S2). The probes were 25~35-mer oligonucleotides with a sequence complementary to the major alleles of the tag SNPs. To prevent elongation of the probe itself, the probes used with *MAP1LC3A*, *MAP1LC3B*, *ATG4A*, *ATG4B*, and *ATG4C* had an amino group modification of the 3′ terminus, and probes for *ATG7* and *ATG13* had a mismatched base added to the 3′ terminus.

The PCR products were subjected to the HRM reaction using the LightCycler480 Instrument (Roche Diagnostics, Basel, Switzerland). The HRM conditions were as follows: thermal denaturation treatment for 1 min at 95 °C and re-treatment for 1 min at 40 °C, followed by altered fluorescence accompanying temperature changes matched to the respective conditions. The probe melting curves were analyzed using the LightCycler480 Gene-Scanning software version 1.5, and the gene polymorphisms were determined. Arbitrarily selected samples were analyzed via PCR-direct DNA sequencing, and the accuracy was determined.

#### 2.2.2. PCR-Restriction Fragment Length Polymorphism (PCR-RFLP) Method

The respective polymorphic regions were amplified by means of PCR. A mixture of genome DNA (10 ng), 1X Go Taq^®^ Colorless Master Mix (Promega, Madison, WI, USA), and forward and reverse primers (0.6 μM each) was prepared to attain a PCR reaction fluid volume of 20 μL. Following denaturation for 2 min at 95 °C, the cycle reactions (30 s at 95 °C, 30 s at an annealing temperature of each primer, and 30 s of elongation at 72 °C) were performed for the number of cycles for each primer, followed by elongation for 5 min at 72 °C (Table S1).

After amplification, the PCR products were confirmed via electrophoresis. The 2% ME agarose gel (Nacalai Tesque, Tokyo, Japan) and Tris–borate–EDTA (TBE) buffer were employed as the phoresis buffer. A Mupid-2plus mini gel phoresis tank (Advance Co., Ltd., Tokyo, Japan) was used as the phoresis tank device. The gel was stained in advance with the Redsafe^TM^ Nucleic Acid Staining Solution (iNtRON Biotechnology, Inc., Seongnam-ci, Republic of Korea). After electrophoresis, the bands were detected under UV illumination using a 2UV High-Performance Transilluminator (UVP, Upland, CA, USA).

Subsequently, the remaining PCR products were fragmented with a restriction enzyme (Table S2). Following fragmentation with the restriction enzyme, electrophoresis was performed using 2% ME agarose gel. The Mupid-2plus mini gel phoresis tank was used with the ME agarose gel, and the TBE buffer was employed as the phoresis buffer. After completion of the electrophoresis, the bands were detected using the 2UV High-Performance Transilluminator to determine the gene polymorphisms.

#### 2.2.3. PCR-Direct DNA Sequencing Method

The respective polymorphic regions were amplified using PCR performed with the same reaction fluid composition and conditions as the PCR-RFLP method. After the PCR, to deactivate the dNTPs and PCR primer, Exonuclease I (Epicentre, Edgewood, MD, USA) and Shrimp Alkaline Phosphatase (Affymetrix, Inc., Sunnyvale, CA, USA) were added to 5 µL of PCR products and enzymatically reacted for 20 min at 37 °C. Then, template DNA was prepared via deactivation for 20 min at 80 °C. Subsequently, the cycle-sequencing reaction was performed according to the protocol of the BigDye^®^ Terminator v3.1 Cycle Sequencing Kit (Life Technologies). Distilled water was added to 25 ng of template DNA, sequencing buffer, and forward or reverse primer (0.1 μM) to attain a total reaction fluid of 10 µL. The hot-start reaction was performed for 30 s at 96 °C; after 25 cycles (10 s at 96 °C, 5 s at 50 °C, and 4 min at 60 °C), elongation was performed for 4 min at 60 °C. The reaction solution was refined using Sephadex G-50 superfine columns (GE Healthcare, Chicago, IL, USA) and dried, followed by the addition of 15 μL Hi-Di formamide (Life Technologies). After denaturation for 2 min at 95 °C, the mixture was placed on ice for ˃5 min, and capillary electrophoresis was performed using an ABI PRISM 3130xl (Life Technologies) to determine the DNA base sequence.

## 3. Results

### 3.1. Clinical Characteristics of the Subjects

As mentioned in previous reports [18,19,20], 94 participants were divided into the AG group, and the average age of the AG group was older than that of the non-AG group (59.2 ± 9.52 vs. 54.9 ± 10.93, *p* = 0.002). On the other hand, there was no difference in gender (male/female) (37/57 vs. 50/56, *p* = 0.266).

### 3.2. HWE

The HWE was not satisfied in rs2144956 of MAP1LC3A, rs807185, and rs2064238 of ATG4A, rs7513520 of ATG4C, and rs6442259 of ATG7. Hence, these SNPs were excluded from the subsequent analysis. The frequencies of all the other SNPs satisfied the HWE.

### 3.3. Results of Correlation Analysis of Genotype and Disease

#### 3.3.1. Correlation Analysis of Polymorphism and AG Progression

Table 1 presents the analysis results of the allele model, the minor allele dominant model, and the minor allele recessive model for the tag SNPs analyzed in the present study. Some results concerning the HRM of the rs4684787 are shown in Supplementary Figure S1 (Figure S1). Considering the rs4684787 of ATG7, the T allele frequency in the AG group was significantly lower than in the non-AG group (*p* = 0.043, odds ratio = 0.657, 95%CI = 0.438–0.987). In addition, the frequencies of the minor allele dominant of rs4684787 (The C/T or T/T genotypes) were significantly lower in the AG group than those in the non-AG group (*p* = 0.034, odds ratio = 0.535, 95%CI = 0.300–0.957). In other words, the C/C genotype of rs4684787 located within the ATG7 demonstrated an increased susceptibility to AG, approximately 2.9 times higher than the alternative genotypes. Conversely, other than rs4684787, no SNP satisfying the HWE exhibited a significant difference in all three analysis models.

#### 3.3.2. Verification of Independence for Tag SNPs

Since there was a significant difference in age between the AG and non-AG groups, multivariate logistic regression analysis was conducted with the rs4684787 of ATG7 and age between the AG and non-AG groups. As shown in Table 2, the C/C genotype of the rs4684787 in ATG7 and age were independently involved in the progression of AG.

## 4. Discussion

The present study, for the first time, suggests that patients with the C/C genotype for the rs4684787 of *ATG7* are more likely to progress to atrophic gastritis than those with the C/T or T/T genotype. Moreover, the multivariate logistic regression analysis revealed that the rs4684787 of *ATG7* and age independently contributed to AG.

According to Correa’s cascade [21], AG is a precancerous lesion that leads to gastric cancer, which is the fifth most common cancer and the third most common cause of cancer mortality [22] and is mainly caused by *H. pylori* infection. Infection with *H. pylori* occurs in approximately half of the world’s population [23]. The infections typically occur in childhood [15], and an infected stomach may develop into AG in adulthood [24]. However, the situations of some children, including a Japanese eight-month- old infant [16], who have AG indicate the host’s genetic factors seem to be involved in the progression of AG. Therefore, detecting the biomarkers and factors that drive the progression of the AG is important.

In the Guideline, which is an official statement from the European Society of Gastrointestinal Endoscopy (ESGE), the European Helicobacter and Microbiota Study Group (EHMSG), the European Society of Pathology (ESP), and the Sociedade Portuguesa de Endoscopia Digestiva (SPED), the Operative Link on Gastritis Assessment (OLGA), and Operative Link on Gastritis Assessment based on Intestinal Metaplasia (OLGIM) systems were proposed for staging of atrophy and/or IM [25]. These systems necessitate histological samples from the gastric body and the antrum/incisura.

However, in the current research, histological biopsy was not undertaken. Instead, the ABC method was employed for the gradation of the AG severity. This method is widely recognized in Japan and includes serological evaluation of *H. pylori* antibodies (Hp) and PG I and II levels. According to this system, individuals with serum PG I ≤ 70 and a PG I/II ratio ≤ 3.0 were categorized as PG(+) [26]. “The patients are stratified into four categories: Group A [Hp(−)PG(−)], noninfected cases; Group B [Hp(+) PG(−)], free or mild chronic atrophic gastritis (CAG) with active or acute *H. pylori* infection; Group C [Hp(+) PG(+)], CAG with chronic inactive *H. pylori* infection; and Group D [Hp(−)PG(+)], severe CAG with extensive IM and spontaneous diminishment of *H. pylori* infection [26]”. It is postulated that the propensity for gastric cancer is most pronounced in Group D, with a successive decrease in risk through Groups C, B, and A [26]. In this investigation, all the subjects were Hp(+); thus, those with PG(+) were classified as severe AG, whereas those with PG(-) were considered as non-severe AG. Further studies employing the OLGA/OLGIM systems are required to refine these findings.

The cytotoxin-associated gene A (CagA), which *H. pylori* injects into the gastric epithelial cell, is one of the virulence factor associated with gastric cancer [27] and AG [28]. On the other hand, the CagA is degraded by the host’s autophagy as a host defense. In the autophagy system, the *ATG8* (*LC3*) conjugation system and the ATG12–ATG16 system are concerned with expanding the double membrane of the phagophore. *Atg7* belongs to the *ATG8* conjugation system and is an essential gene for autophagy. A deletion of the Atg7 gene in neurons suppresses autophagy in mice.

The Rs4684787 in *ATG7* is in the intron region of ATG7. Genetic polymorphisms in the intron region could impact the splicing function and regulatory functions of genes. There is just one report concerning the rs4684787 in *ATG7*. In that report, Lei Xia et al. reported that the rs4684787 in *ATG7* showed no significance between cerebral palsy in Chinese infants and controls [29]. According to the GTEx portal, a comprehensive correlation database of gene expression and SNPs in various tissues, it has been reported that the C/C genotype of rs4684787 does not change the ATG7 expression in the stomach when compared with cases with the C/T or T/T genotype. However, this genotype is linked to increased expression of *ATG7* introns in skeletal muscle, while decreased expression is observed in cultured fibroblast cells and skin that have been exposed to the sun, relative to the C/T or T/T genotypes. Although no functional analysis of the rs4684787 in *ATG7* has been performed, the C/C genotype of rs4684787 may influence the function of autophagy. That is, when patients with the C/C genotype rs4684787 in *ATG7* are infected with *H. pylori*, the patient’s autophagy function might be attenuated, and insufficient CagA degradation might occur in gastric mucosal cells. Consequently, CagA-induced proinflammatory cytokines, interleukin (IL)-1, IL-6, IL-18, tumor necrosis factor α (TNFα), and interferon-γ (IFN-γ) [30,31,32], will increase, triggering gastric mucosa inflammation and atrophic gastritis.

There are several limitations in this study. First, the sample size in this study was small. Furthermore, we did not examine the validation study. Second, *H. pylori* staining and the type of CagA were not examined, although almost all the Japanese patients are infected with East-Asian-type *H. pylori* strain. Third, a functional analysis of the rs4684787 in *ATG7* needs to be performed. Fourth, a quantitative trait loci study of rs4684787 was not conducted. Fifth, biopsies were not conducted in this study. AG in this study was not evaluated using the OLGA/OLGIM systems. Sixth, the cohort was exclusively composed of Japanese individuals; therefore, it is necessary to ascertain whether the polymorphism rs4684787 in the *ATG7* gene is indicative of AG progression in populations of different ethnicities. Future research evaluating with the OLGA/OLGIM systems should encompass a more extensive cohort, inclusive of diverse ethnic groups. However, this study could detect the biomarker of the progression of AG. The biomarker could help encourage the patients likely to develop severe AG to undergo timely endoscopic screening.

## 5. Conclusions

The C/C genotype of the rs4684787 in *ATG7* could be an age-independent biomarker for predicting the Japanese patients infected with *H. pylori* whose AG is likely to progress. This finding helps stratify the patients who need timely endoscopic screening or who need early eradication of *H. pylori*, even if the patients are children. In the future, we hope that further functional analysis of the rs4684787 in *ATG7* will lead to pathological findings and the development of new therapeutic GMA drugs to reduce gastric cancer risk.

## Figures and Tables

**Table 1:** Allele and genotype comparisons between the with and without AG groups in three genetic models.

Gene	SNP	Genotype	Number of		Genetic Model	OR (95%CI)	*p* Value *
			AG *n* = 94 (%)	non-AG *n* = 106 (%)			
*MAP1LC3A*	rs2424994	C/C	73 (77.7)	89 (84.0)	Allele model	1.346 (0.704–2.574)	0.367
		C/T	20 (21.3)	15 (14.2)	Dominant model	1.506 (0.740–3.065)	0.257
		T/T	1 (1.1)	2 (1.9)	Recessive model	0.559 (0.050–6.268)	1.000
	rs6059909	C/C	33 (35.1)	43 (40.6)	Allele model	1.086 (0.723–1.632)	0.692
		C/A	51 (54.3)	50 (47.2)	Dominant model	1.262 (0.711–2.240)	0.427
		A/A	10 (10.6)	13 (12.3)	Recessive model	0.852 (0.355–2.044)	0.719
*MAP1LC3B*	rs9903	C/C	34 (36.2)	40 (37.7)	Allele model	1.093 (0.730–1.637)	0.667
		C/T	46 (48.9)	53 (50.0)	Dominant model	1.070 (0.602–1.902)	0.819
		T/T	14 (14.9)	13 (12.3)	Recessive model	1.252 (0.556–2.820)	0.587
	rs8044820	A/A	57 (60.6)	59 (55.7)	Allele model	1.003 (0.639–1.564)	0.989
		A/G	26 (27.7)	40 (37.7)	Dominant model	0.815 (0.464–1.432)	0.477
		G/G	11 (11.7)	7 (6.6)	Recessive model	1.874 (0.695–5.052)	0.209
*ATG4B*	rs4675930	G/G	53 (56.4)	67 (63.2)	Allele model	1.274 (0.789–2.059)	0.321
		G/A	38 (40.4)	37 (34.9)	Dominant model	1.329 (0.754–2.344)	0.326
		A/A	3 (3.2)	2 (1.9)	Recessive model	1.714 (0.280–10.488)	0.667
	rs6709768	G/G	63 (67.0)	76 (71.7)	Allele model	1.113 (0.654–1.893)	0.694
		G/C	30 (31.9)	27 (25.5)	Dominant model	1.247 (0.682–2.278)	0.473
		C/C	1 (1.1)	3 (2.8)	Recessive model	0.369 (0.038–3.611)	0.624
	rs4247179	T/T	71 (75.5)	69 (65.1)	Allele model	0.618 (0.354–1.080)	0.089
		T/C	23 (24.5)	35 (33.0)	Dominant model	0.604 (0.326–1.120)	0.108
		C/C	0 (0.0)	2 (1.9)	Recessive model	0.221 (0.011–4.669)	0.499
*ATG4C*	rs11580642	G/G	43 (45.7)	46 (43.4)	Allele model	1.054 (0.699–1.591)	0.801
		G/A	35 (37.2)	47 (44.3)	Dominant model	0.909 (0.520–1.590)	0.739
		A/A	16 (17.0)	13 (12.3)	Recessive model	1.468 (0.665–3.238)	0.340
*ATG7*	rs346078	G/G	50 (53.2)	45 (42.5)	Allele model	0.739 (0.481–1.136)	0.168
		G/C	37 (39.4)	51 (48.1)	Dominant model	0.649 (0.371–1.135)	0.129
		C/C	7 (7.5)	10 (9.4)	Recessive model	0.772 (0.282–2.118)	0.615
	rs9818393	C/C	71 (75.5)	77 (72.6)	Allele model	0.896 (0.508–1.580)	0.703
		C/T	21 (22.3)	27 (25.5)	Dominant model	0.860 (0.456–1.623)	0.642
		T/T	2 (2.1)	2 (1.9)	Recessive model	1.130 (0.156–8.187)	1.000
	rs4684073	A/A	39 (41.5)	46 (43.4)	Allele model	0.940 (0.621–1.422)	0.769
		A/G	47 (50.0)	46 (43.4)	Dominant model	1.081 (0.616–1.897)	0.785
		G/G	8 (8.5)	14 (13.2)	Recessive model	0.611 (0.244–1.529)	0.289
	rs4684787	C/C	42 (44.7)	32 (30.2)	Allele model	0.657 (0.438–0.987)	0.043
		C/T	41 (43.6)	56 (52.8)	Dominant model	0.535 (0.300–0.957)	0.034
		T/T	11 (11.7)	18 (17.0)	Recessive model	0.648 (0.289–1.453)	0.29
	rs2305295	T/T	66 (70.2)	71 (67.0)	Allele model	0.830 (0.499–1.381)	0.473
		T/C	24 (25.5)	28 (26.4)	Dominant model	0.861 (0.473–1.567)	0.623
		C/C	4 (4.3)	7 (6.6)	Recessive model	0.629 (0.178–2.219)	0.467
	rs14016	C/C	40 (42.6)	44 (41.5)	Allele model	0.971 (0.645–1.462)	0.887
		C/T	41 (43.6)	47 (44.3)	Dominant model	0.958 (0.546–1.682)	0.881
		T/T	13 (13.8)	15 (14.2)	Recessive model	0.974 (0.437–2.169)	0.948
	rs9849485	C/C	35 (37.2)	45 (42.5)	Allele model	1.134 (0.755–1.704)	0.545
		C/T	46 (48.9)	47 (44.3)	Dominant model	1.244 (0.704–2.196)	0.452
		T/T	13 (13.8)	14 (13.2)	Recessive model	1.055 (0.468–2.375)	0.898
	rs2248971	C/C	39 (41.5)	52 (49.1)	Allele model	1.195 (0.786–1.817)	0.404
		C/T	45 (47.9)	43 (40.6)	Dominant model	1.358 (0.776–2.377)	0.284
		T/T	10 (10.6)	11 (10.4)	Recessive model	1.028 (0.416–2.542)	0.952
	rs2454511	G/G	38 (40.4)	51 (48.1)	Allele model	1.113 (0.730–1.696)	0.619
		G/A	50 (53.2)	45 (42.05)	Dominant model	1.367 (0.780–2.395)	0.275
		A/A	6 (6.4)	10 (9.4)	Recessive model	0.655 (0.228–1.875)	0.427
	rs2880897	G/G	33 (35.1)	47 (44.3)	Allele model	1.057 (0.701–1.593)	0.792
		G/A	54 (57.4)	44 (41.5)	Dominant model	1.473 (0.832–2.607)	0.183
		A/A	7 (7.4)	15 (14.2)	Recessive model	0.488 (0.190–1.255)	0.130
	rs9840982	C/C	83 (88.3)	83 (78.3)	Allele model	0.582 (0.287–1.179)	0.129
		C/A	9 (9.6)	22 (20.8)	Dominant model	0.478 (0.219–1.044)	0.060
		A/A	2 (2.1)	1 (0.9)	Recessive model	2.283 (0.204–25.587)	0.492
	rs2437681	T/T	48 (51.1)	62 (58.5)	Allele model	1.113 (0.707–1.751)	0.644
		T/C	43 (45.7)	37 (34.9)	Dominant model	1.350 (0.772–2.362)	0.292
		C/C	3 (3.2)	7 (6.6)	Recessive model	0.466 (0.117–1.857)	0.269
*ATG13*	rs6485686	T/T	82 (87.2)	86 (81.1)	Allele model	0.732 (0.361–1.484)	0.385
		T/G	10 (10.6)	19 (17.9)	Dominant model	0.629 (0.289–1.369)	0.240
		G/G	2 (2.1)	1 (0.9)	Recessive model	2.283 (0.204–25.587)	0.602
	rs10838611	G/G	47 (50.0)	58 (54.7)	Allele model	1.091 (0.701–1.700)	0.699
		G/C	42 (44.7)	41 (38.7)	Dominant model	1.208 (0.693–2.108)	0.505
		C/C	5 (5.3)	7 (6.6)	Recessive model	0.795 (0.244–2.593)	0.703
	rs13448	C/C	41 (43.6)	46 (43.4)	Allele model	0.948 (0.622–1.444)	0.803
		C/T	47 (50.0)	51 (48.1)	Dominant model	0.991 (0.566–1.735)	0.975
		T/T	6 (6.4)	9 (8.5)	Recessive model	0.735 (0.251–2.148)	0.572
	rs76470208	A/A	58 (61.7)	67 (63.2)	Allele model	1.032 (0.637–1.671)	0.898
		A/T	32 (34.0)	34 (32.1)	Dominant model	1.066 (0.601–1.892)	0.826
		T/T	4 (4.3)	5 (4.7)	Recessive model	0.898 (0.234–3.446)	1.000
	rs76533702	T/T	39 (41.5)	43 (40.6)	Allele model	0.984 (0.657–1.473)	0.938
		T/C	38 (40.4)	44 (41.5)	Dominant model	0.963 (0.547–1.693)	0.895
		C/C	17 (18.1)	19 (17.9)	Recessive model	1.011 (0.491–2.082)	0.977

* Alleles and genotypes in the three genetics models were compared using the chi-square or Fisher’s exact test.

**Table 2:** The results of the multivariate logistic regression analysis with C/C genotype of the rs4684787 in ATG7 and age between the AG and non-AG groups.

Factor	OR (95%CI)	*p* Value
C/C genotype of rs4684787 in *ATG7*	1.898 (1.048–3.437)	0.034
Age	1.043 (1.013–1.073)	0.004

## Data Availability

The datasets from this study are not publicly available due to such access not being included in the informed consent.

## Supplementary Materials

The following supporting information can be downloaded at https://www.mdpi.com/article/10.3390/jcm13020629/s1, Table S1: Information regarding the tag SNP genotyping; Table S2: Information regarding the probe-based HRM and RFLP; Figure S1: The result of the HRM analysis of the single nucleotide polymorphism rs4684787 in some patients.

## References

[1] McEwan D.G., Dikic I. (2010). Not all autophagy membranes are created equal. Cell.

[2] Dikic I., Elazar Z. (2018). Mechanism and medical implications of mammalian autophagy. Nat. Rev. Mol. Cell Biol..

[3] Thomas D.R., Newton P., Lau N., Newton H.J. (2020). Interfering with Autophagy: The Opposing Strategies Deployed by *Legionella pneumophila* and *Coxiella burnetii* Effector Proteins. Front. Cell Infect. Microbiol..

[4] Mizushima N., Levine B. (2020). Autophagy in Human Diseases. N. Engl. J. Med..

[5] Klionsky D.J., Petroni G., Amaravadi R.K., Baehrecke E.H., Ballabio A., Boya P., Bravo-San Pedro J.M., Cadwell K., Cecconi F., Choi A.M.K. (2021). Autophagy in major human diseases. EMBO J..

[6] Rabinowitz J.D., White E. (2010). Autophagy and metabolism. Science.

[7] Grosjean I., Roméo B., Domdom M.A., Belaid A., D’Andréa G., Guillot N., Gherardi R.K., Gal J., Milano G., Marquette C.H. (2022). Autophagopathies: From autophagy gene polymorphisms to precision medicine for human diseases. Autophagy.

[8] Wu M., Chen B., Pan X., Su J. (2020). Prognostic Value of Autophagy-related Proteins in Human Gastric Cancer. Cancer Manag. Res..

[9] Rugge M., Correa P., Dixon M.F., Fiocca R., Hattori T., Lechago J., Leandro G., Price A.B., Sipponen P., Solcia E. (2002). Gastric mucosal atrophy: Interobserver consistency using new criteria for classification and grading. Aliment. Pharmacol. Ther..

[10] Barrozo R.M., Hansen L.M., Lam A.M., Skoog E.C., Martin M.E., Cai L.P., Lin Y., Latoscha A., Suerbaum S., Canfield D.R. (2016). CagY Is an Immune-Sensitive Regulator of the Helicobacter pylori Type IV Secretion System. Gastroenterology.

[11] Sitarz R., Skierucha M., Mielko J., Offerhaus G.J.A., Maciejewski R., Polkowski W.P. (2018). Gastric cancer: Epidemiology, prevention, classification, and treatment. Cancer Manag. Res..

[12] Masuyama H., Yoshitake N., Sasai T., Nakamura T., Masuyama A., Zuiki T., Kurashina K., Mieda M., Sunada K., Yamamoto H. (2015). Relationship between the degree of endoscopic atrophy of the gastric mucosa and carcinogenic risk. Digestion.

[13] Lee S.Y. (2016). Endoscopic gastritis, serum pepsinogen assay, and Helicobacter pylori infection. Korean J. Intern. Med..

[14] Fujiwara Y., Watanabe T., Muraki M., Yamagami H., Tanigawa T., Shiba M., Tominaga K., Arakawa T. (2015). Association between chronic use of proton pump inhibitors and small- intestinal bacterial overgrowth assessed using lactulose hydrogen breath tests. Hepatogastroenterology.

[15] Ricuarte O., Gutierrez O., Cardona H., Kim J.G., Graham D.Y., El-Zimaity H.M. (2005). Atrophic gastritis in young children and adolescents. J. Clin. Pathol..

[16] Kakiuchi T., Nakayama A., Shimoda R., Matsuo M. (2019). Atrophic gastritis and chronic diarrhea due to Helicobacter pylori infection in early infancy: A case report. Medicine.

[17] Honma H., Nakayama Y., Kato S., Hidaka N., Kusakari M., Sado T., Suda A., Lin Y. (2019). Clinical features of Helicobacter pylori antibody-positive junior high school students in Nagano Prefecture, Japan. Helicobacter.

[18] Yamaguchi N., Sakaguchi T., Isomoto H., Inamine T., Ueda H., Fukuda D., Ohnita K., Kanda T., Kurumi H., Matsushima K. (2023). ATG16L1 and ATG12 Gene Polymorphisms Are Involved in the Progression of Atrophic Gastritis. J. Clin. Med..

[19] Yamaguchi N., Sakaguchi T., Isomoto H., Inamine T., Tsukamoto R., Fukuda D., Ohnita K., Kanda T., Matsushima K., Hirayama T. (2023). Polymorphism in autophagy-related genes LRP1 and CAPZA1 may promote gastric mucosal atrophy. Genes Environ..

[20] Isomoto H., Sakaguchi T., Inamine T., Takeshita S., Fukuda D., Ohnita K., Kanda T., Matsushima K., Honda T., Sugihara T. (2022). SNP rs2920280 in PSCA Is Associated with Susceptibility to Gastric Mucosal Atrophy and Is a Promising Biomarker in Japanese Individuals with Helicobacter pylori Infection. Diagnostics.

[21] Lahner E., Conti L., Annibale B., Corleto V.D. (2020). Current Perspectives in Atrophic Gastritis. Curr. Gastroenterol. Rep..

[22] Sung H., Ferlay J., Siegel R.L., Laversanne M., Soerjomataram I., Jemal A., Bray F. (2021). Global Cancer Statistics 2020: GLOBOCAN Estimates of Incidence and Mortality Worldwide for 36 Cancers in 185 Countries. CA Cancer J. Clin..

[23] Yamaoka Y. (2010). Mechanisms of disease: Helicobacter pylori virulence factors. Nat. Rev. Gastroenterol. Hepatol..

[24] Aguilera Matos I., Diaz Oliva S.E., Escobedo A.A., Villa Jiménez O.M., Velazco Villaurrutia Y.D.C. (2020). Helicobacter pylori infection in children. BMJ Paediatr. Open.

[25] Pimentel-Nunes P., Libânio D., Marcos-Pinto R., Areia M., Leja M., Esposito G., Garrido M., Kikuste I., Megraud F., Matysiak-Budnik T. (2019). Management of epithelial precancerous conditions and lesions in the stomach (MAPS II): European Society of Gastrointestinal Endoscopy (ESGE), European Helicobacter and Microbiota Study Group (EHMSG), European Society of Pathology (ESP), and Sociedade Portuguesa de Endoscopia Digestiva (SPED) guideline update 2019. Endoscopy.

[26] Chen X.Z., Huang C.Z., Hu W.X., Liu Y., Yao X.Q. (2018). Gastric Cancer Screening by Combined Determination of Serum Helicobacter pylori Antibody and Pepsinogen Concentrations: ABC Method for Gastric Cancer Screening. Chin. Med. J..

[27] Bessède E., Mégraud F. (2022). Microbiota and gastric cancer. Semin. Cancer Biol..

[28] Herrero R., Heise K., Acevedo J., Cook P., Gonzalez C., Gahona J., Cortés R., Collado L., Beltrán M.E., Cikutovic M. (2020). Regional variations in Helicobacter pylori infection, gastric atrophy and gastric cancer risk: The ENIGMA study in Chile. PLoS ONE.

[29] Xia L., Xu J., Song J., Xu Y., Zhang B., Gao C., Zhu D., Zhou C., Bi D., Wang Y. (2019). Autophagy-Related Gene 7 Polymorphisms and Cerebral Palsy in Chinese Infants. Front. Cell Neurosci..

[30] Hatakeyama M. (2017). Structure and function of Helicobacter pylori CagA, the first-identified bacterial protein involved in human cancer. Proc. Jpn. Acad. Ser. B Phys. Biol. Sci..

[31] Sakamoto H., Yoshimura K., Saeki N., Katai H., Shimoda T., Matsuno Y., Saito D., Sugimura H., Tanioka F., Kato S. (2008). Genetic variation in PSCA is associated with susceptibility to diffuse-type gastric cancer. Nat. Genet..

[32] Yamauchi K., Choi I.J., Lu H., Ogiwara H., Graham D.Y., Yamaoka Y. (2008). Regulation of IL-18 in Helicobacter pylori infection. J. Immunol..

