# Supplementary figures and images for: Autophagy-Related Gene ATG7 Polymorphism Could Potentially Serve as a Biomarker of the Progression of Atrophic Gastritis
Source: J Clin Med. 2024 Jan 22;13(2):629. doi: 10.3390/jcm13020629 (PMC10817077; doi:10.3390/jcm13020629)

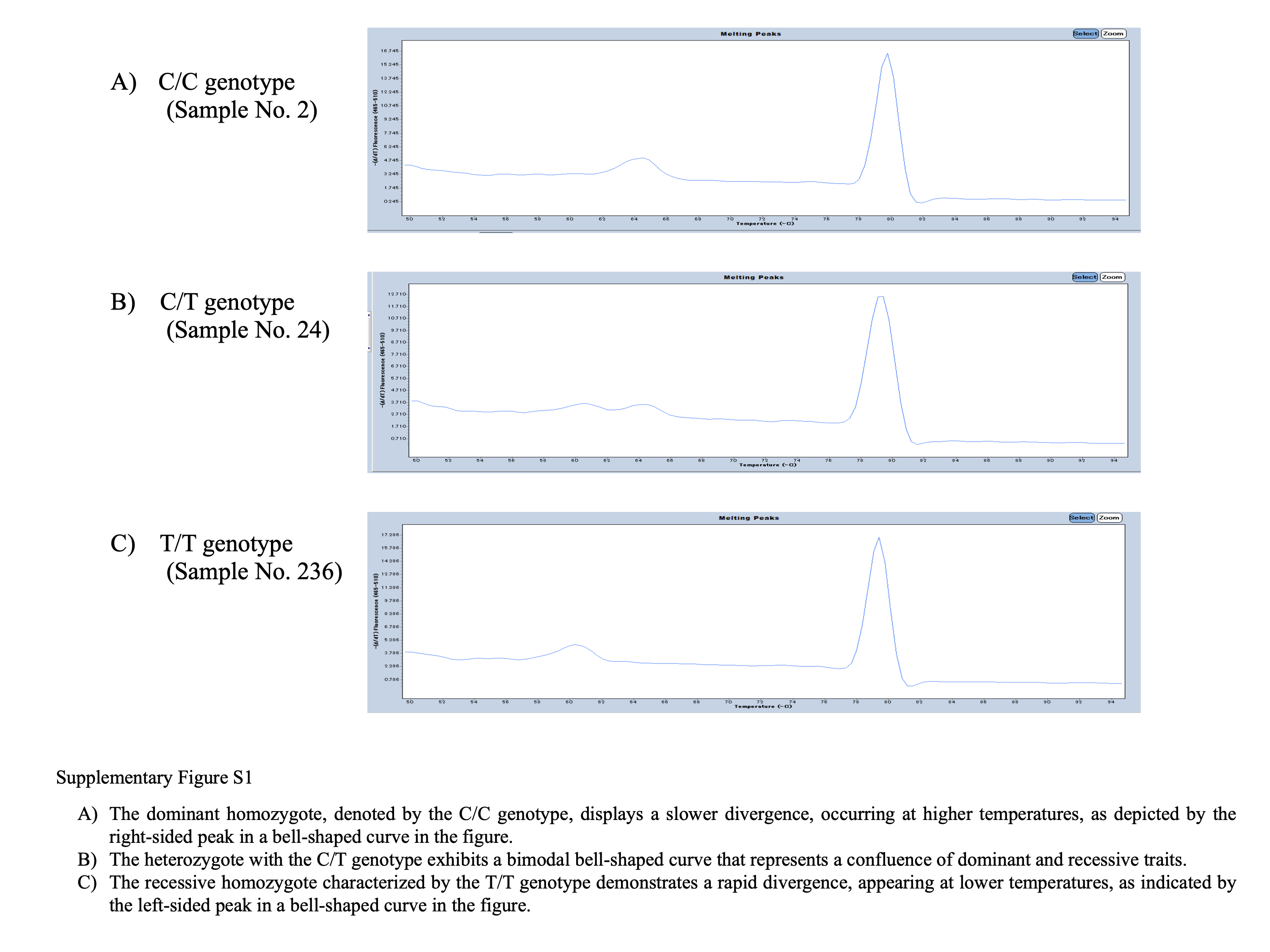

Supplement: Supplementary file 1 [file jcm-13-00629-s001.zip › Figiure S1.tif]
